# DNA testing for sickle cell anemia in Africa: Implementation choices for the Democratic Republic of Congo

**DOI:** 10.1002/jcla.24398

**Published:** 2022-04-11

**Authors:** Mamy Ngole, Valerie Race, Gloire Mbayabo, Paul Lumbala, Cathy Songo, Prosper Tshilobo Lukusa, Koenraad Devriendt, Gert Matthijs, Aimé Lumaka

**Affiliations:** ^1^ Center for Human Genetics Faculty of Medicine KU Leuven Leuven Belgium; ^2^ Center for Human Genetics Faculty of Medicine University of Kinshasa Kinshasa Democratic Republic of Congo; ^3^ Department of Medical Biology Faculty of Medicine University of Kinshasa Kinshasa Democratic Republic of Congo; ^4^ Department of Pediatrics Faculty of Medicine University of Kinshasa Kinshasa Democratic Republic of Congo; ^5^ GIGA‐R Laboratoire de Génétique Humaine University of Liège Liège Belgium

**Keywords:** buccal swab, DNA based‐tests, sickle cell anemia, umbilical cord blood, venous blood

## Abstract

**Background:**

Hemoglobin‐based tests form the reference diagnostic test for SCA. In limited resource countries, these tests face limitations including cost, low sensitivity due to recurrent transfusions in endemic malaria region, and interference from fetal hemoglobin in neonatal diagnostic. This study aimed at adapting DNA‐based SCA tests to limited resource countries and evaluating the economic benefit.

**Methods:**

338 participants were recruited in the Democratic Republic of Congo, sorted in 3 cohorts based on venous blood, umbilical cord blood (UCB) and buccal swab sampling. RFLP was performed to identify mutated allele. The feasibility and technical validity of this RFLP was evaluated for specimens collected on DBS cards and on EDTA tubes. RFLP on DBS stored at room temperature was regularly repeated to assess sample conservation. Finally, the cost analysis was performed.

**Results:**

DBS cards yielded identical results to extracted DNA. Repeated testing returned the same result after four years. The DBS‐based test performed on UCB or on buccal swab had a sensitivity and a precision of 100%. Cost comparison indicated that our approach costs half price of the widely used isoelectrofocussing of hemoglobin.

**Conclusion:**

The implemented DNA‐based test approach overcomes the limitations faced by hemoglobin‐based tests, while being more affordable. We propose to implement the RFLP test as a first line diagnostic test after transfusion and as second tiers for newborn screening. However, users should be aware that this test is unable to differentiate HbC from HbS or identify other point mutation of gene deletion of HBB gene.

## Graphical abstract

The DNA‐based test for SCA using DBS is efficient on all three types of biologic samples and costs half cheaper than the widely used hemoglobin electrophoresis method. Moreover, repeated testing on DBS stored at local conditions returned the same results after 4 years. Due to its technical validity and economic benefits, this DNA‐based SCA test appears to be suited to resource‐limited settings.

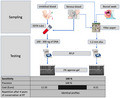

## 1 INTRODUCTION

Low‐income countries such as the Democratic Republic of Congo (DRC) suffer a heavy burden of sickle cell anemia (SCA) with about 2% of newborns being homozygous for the classical SCA mutation, and 17%–24% being heterozygous carriers.^1^, ^2^ The disease‐related mortality remains high in many sub‐Saharan African countries as 50% to 90% of affected children die before 5 years of age,^3^ often before being formally diagnosed. Thus far, nationwide newborn screening is not available in DRC.

The most commonly available SCA tests in many localities in DRC include the Itano solubility test and the Emmel sickling test. These tests detect only the presence of hemoglobin from the abnormal allele (HbS) and are unable to detect the hemoglobin from the reference allele (HbA). Therefore, these tests are unable to distinguish sickle cell trait (AS profile from heterozygotes individuals) from SCA (SS profile from homozygotes patients).^4^, ^5^ Thus, they are of limited diagnostic and clinical value. Electrophoresis on acetate cellulose and isoelectrofocussing (IEF) has been introduced in DRC since 1954 and 2009, respectively.^1^, ^6^ However, these technologies are not widely available across the country. Samples need to be shipped from rural areas to the laboratories located in urban areas. Such sample transfer and storage represent big challenges and may impact on the cost and quality of the test. These technical and logistic challenges as well as the cost of the test should be considered when implementing a diagnostic test for SCA in a resource‐limited setting.

In addition, the hemoglobin electrophoresis‐based techniques have low accuracy during the neonatal period because of the presence of fetal hemoglobin, or after a transfusion because of the presence of donor's hemoglobin.^7^ Studies on Congolese SCA patients have reported that anemia is the most common sign and often the first manifestation.^8^, ^9^ Thus, when SCA is suspected, patients have usually a recent history of transfusion, forcing practitioners to delay the diagnostic test for 3 months. Unfortunately, because of other intercurrent factors, such as malaria and other infections, that are very frequent in this setting, it is common for these children to return to the hospital and receive another transfusion within these 3 months’ window, further delaying SCA confirmation by hemoglobin electrophoresis‐based methods. Therefore, the optimal diagnostic test for SCA should retain high accuracy regardless of recent transfusion or in neonatal period.

Previous studies have shown that DNA‐based tests are not affected by transfusion or fetal hemoglobin.^10^ Thus, DNA‐based tests would be preferable for a resource‐limited setting. However, the implementation of such a technique has to address important challenges for sample collection, transfer, and cold chain requirements for storage, as well as the test accuracy in the neonatal period and following a recent transfusion. This study aimed to evaluate the technical validity of a DNA‐based SCA diagnostic test suited for DRC in particular and for low‐income settings in general.

## 2 METHODS

### 2.1 Study design

This study was conducted in three cohorts with different biological specimen types (venous blood, umbilical cord blood, and saliva) in each phase.

#### 2.1.1 Cohort 1. Venous blood

##### Study population

A cohort of 166 participants was recruited, both at the Saint Luc Hospital and the Centre de Médecine Mixte et d’Anémie SS (CMMASS), respectively, in Kisantu (Kongo Central Province) and Kinshasa (the capital city) in the DRC. These patients were previously diagnosed as homozygous (SS; n = 145), heterozygous (AS; n = 12), and wild‐type (AA; n = 9) using a hemoglobin electrophoresis‐based technique. The three hemoglobin types represent the three genotypes for the SCA mutation. The SS individuals are SCA patients, AS are heterozygous carriers while the AA are homozygous wild‐type individuals.

##### Samples collection and transportation

**FIGURE 1:**
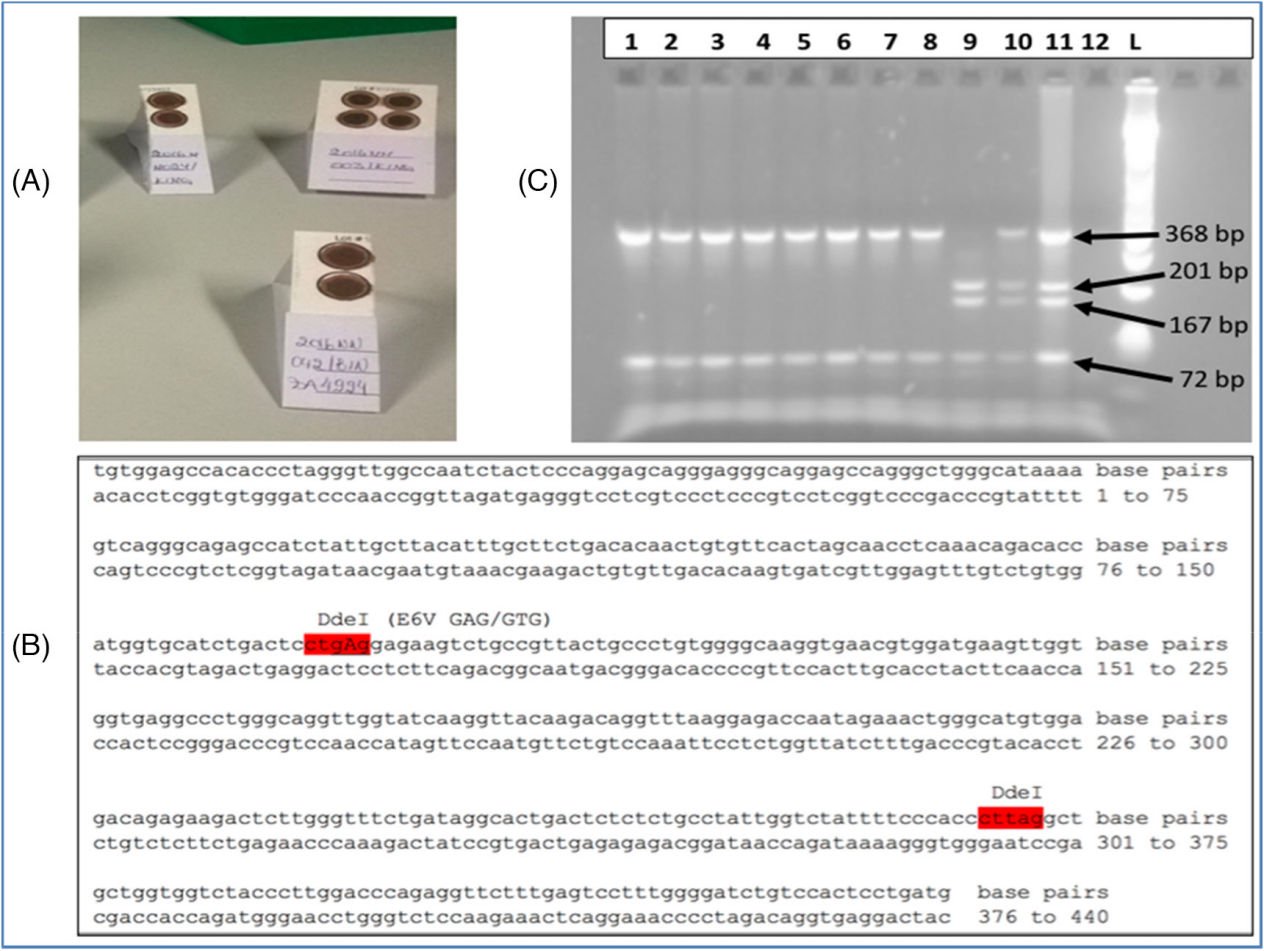
Sample collection, experimental design, and interpretation. A, Blood sample spotted onto FTA elute cards. Here are illustrated the full FTA card and two pieces of a split FTA card, all spotted with different samples. B, The figure shows the target PCR sequence. The two restriction sites sequences are underlined with a dashed line. The S mutation is located on the first restriction site. C, Pattern of restriction products on 2% agarose gel. Lane 1 to 8 shows homozygous mutation SS profiles, lane 9 shows homozygous wild type AA, lane 10 illustrates heterozygous AS, lane 11 contains a positive control, whereas lane 12 is the no DNA. The size ladder is on the L lane

For each participant, 4 ml of peripheral venous blood was collected in a EDTA‐coated tube (BD Diagnostics). Before removing the sampling needle, a drop of blood (approximately 50 µl of blood) was deposited on a circle of a FTA (Flinders Technology Associates) Elute card (Whatman WB120206). This type of FTA card is chemically treated filter papers that lyse cells, denature proteins and stabilize nucleic acids on the cellulose fibers (Figure 1A). The FTA cards have four circular areas where the sample should be deposited. More details about the FTA Elute card are provided by the manufacturer: at: https://webshop.fishersci.com/webfiles/fr/web/FS2013/EU_FR_13LAB_P0167.pdf?_ga=2.10467506.685819103.1647441468‐337705928.1647441468

The spotted FTA cards were kept at room temperature for 90 minutes allowing the sample to dry prior to transportation in an individual plastic bag, adequately labeled. In order to reduce the cost of the experiment, each individual FTA card was divided into two pieces, allowing one card to be used for two patients (Figure 1A). The spotted FTA cards (dried blood spot, DBS) were stored in zip bags at room temperature on a clean shelve until being used for applications as further described below.

Blood samples in EDTA tubes were transported in isotherm boxes from the sampling site to the laboratory of the Center for Human Genetics of the University of Kinshasa (UNIKIN, www.coshg.org).

##### DNA extraction and molecular test

The venous blood collected in EDTA tubes was used for DNA extraction by salt‐induced precipitation of cellular protein method also known as standard salting out procedure.^11^ The extracted DNA was then re‐suspended in 300 µl of TE buffer and kept at −20°C until the analysis. The standard manual salting out extraction procedure lasts around 24 hours. In our study, DNA extraction was performed within two days following the sample collection.

A restriction fragment length polymorphism (RFLP) test for SCA was implemented in our laboratory as previously described.^12^ This test uses the DdeI restriction enzyme to differentiate between the mutant allele (S) and the wild‐type allele (A) on a PCR product spanning the SCA mutation site (Figure 1B).

#### 2.1.2 *Cohort* *2. Umbilical cord blood*

##### Study population

Umbilical blood cord was collected from 102 newborns in two maternities in Kinshasa, the Maternity Hospital of Binza (MHB) in the north of Kinshasa, and the Maternity Hospital of Kingasani (MHK) in the east of Kinshasa.

##### Samples collection and transportation

Umbilical cord blood (UCB) was obtained “in utero,” meaning before the placenta was expelled, the fetal extremity of the umbilical cord was disinfected, clamped, and sectioned. Blood sample was collected by gravity into a 4 ml EDTA tube. A drop of UCB was directly spotted onto two circles of a FTA card. Samples were transported to the laboratory of the Center for Human Genetics of the Kinshasa University at room temperature packed in plastic zip bags for spotted FTA cards and in isotherm boxes for UCB in EDTA tubes.

The UCB sample collected in EDTA tubes was used for DNA extraction following the standard salting out procedure, whereas spotted FTA cards were used as described below.

#### 2.1.3 *Cohort* *3. Saliva*

##### Study population

A group of 70 participants was recruited, among which 20 were from the CMMASS and 50 from the University Hospitals of the University of Kinshasa (CUK). Previous molecular diagnosis using DNA extracted from peripheral blood returned homozygous mutation genotype (SS hemoglobin type) in 43, heterozygous (AS hemoglobin type) in 13, and homozygous wild type (AA hemoglobin type) in 14.

##### Samples collection and transportation

For each participant, buccal cells were collected using sterilized cotton buds made with wooden sticks. Patients were recommended not to eat, drink, smoke, chew gum, neither use toothpaste nor mouthwash during the 30 minutes preceding sample collection. Samples were collected by scratching while rotating the swab on the mucosa of cheeks for 30–60 seconds. After sampling, the swab was scratched against a circle of FTA elute card for 10 seconds in order to transfer the cells from the swab onto the FTA card.

##### DNA testing

Each sample collected in EDTA tubes underwent DNA extraction by manual salting‐out method (SO).^11^ Extracted DNA was later used for a PCR reaction and the RFLP using an Applied Biosystems 2720 Thermal Cycler. We refer to this protocol as SO‐RFLP across the article.

For samples collected on FTA cards, a DNA extraction step was not performed. One 1.2 mm disk was punched from the spotted FTA card, washed with purified water in a 2 ml reaction tube as recommended by the manufacturer, then transferred into the PCR reaction tube and mixed with the PCR reaction mix (Table 1, 2). The PCR product was used for enzymatic restriction as described below. In order to prevent cross‐contamination while punching, an unused card was punched three times in between two samples as recommended by the manufacturer. The remainder of the spotted FTA card was stored at room temperature in a zip bag in a dust‐free closet. This second protocol is referred to as FTA‐RFLP in this article.

**TABLE 1:** Composition of the PCR Mix

Solution	Amount per sample
Primer F + R (2.5 µm)	2 µl
Amplification buffer (Roche)	2 µl
dNTP’s 2mM	2 µl
Taq DNA polymerase (5 U/µl) (Roche)	0.1 µl
H_2_O	12.9µl
Total volume	19 µl

The PCR targeted a fragment of 440 bp in the first exon of the hemoglobin beta gene (*HBB*; GRCh38 chr11:5225464‐5229395, Figure 1B) using the following primer pair:

Forward TGTGGAGCCACACCCTAGGGTTG and Reverse CATCAGGAGTGGACAGATCC.

For each run, two controls were used, including a No DNA control (disk punched from blank unspotted paper) and a positive control (disk punched from FTA card spotted with blood sample of a Congolese SCA patient with known genetic profile). The composition of the PCR mix is presented in Table 1.

Either 1 µl of DNA extracted by SO (100–300 ng) or a 1.2 mm washed FTA punch was added into the PCR mix for the SO‐RFLP or FTA‐RFLP, respectively.

The PCR program consisted of an initial denaturation step at 95°C for 5 min, followed by 32 amplification cycles, each cycle included a quick denaturation at 95°C for 30 s, annealing at 58°C for 30 s, and extension at 72°C for 30 s. The reaction was concluded with a final extension step at 72°C for 5 min.

The PCR product was digested using the DdeI enzyme (Promega Corporation, Belgium, Cat: R6291) according to a standard protocol. The DdeI enzyme has two restriction sites (ctgag/) within the PCR product (Figure 1B). The digestion products were visualized on 2% agarose gel (Figure 1C). A non‐mutated or wild‐type allele produced three fragments with 201 bp, 167 bp, and 72 bp size, respectively. Conversely, on an allele with the S mutation (mutated allele), the first restriction site is abolished, resulting in only two fragments of 368 bp and 72 bp, respectively. For homozygotes SS individuals, only these two bands appear, three bands are observed for homozygotes AA, whereas four bands are identified in heterozygotes AS, that is, 368 bp, 167 bp, 201 bp, and 72 bp bands.

##### Sensitivity and precision

Sensitivity, considered as the ability of the test to identify variants that are present in a sample, was calculated using the formula TP/TP + FN. The precision, defined as the fraction of variant calls that match the expected, reflecting the number of FP per test, was determined using the formula TP/TP +FP.^13^ As previously demonstrated, the sensitivity threshold is influenced by the prevalence of the disease in the studied population.^14^ For the previously reported incidence of 1.4% in DRC,^1^ an error rate of 5% will result in multiple children being denied timely care. Therefore, we set the minimum threshold at 99%.

##### Evaluation of repeatability and reproducibility

Repeatability, defined as the percent agreement between the results of successive tests carried out under the same conditions of measurement,^13^ was assessed in two ways: the intra‐run and inter‐run repeatability. Five FTA cards spotted with venous blood were randomly selected. Three disks from each in five cards were included a run to evaluate intra‐run repeatability. This was repeated three times to assess inter‐run repeatability.

We also assessed the reproducibility, which evaluates whether changes in testing platforms, reagent supplies, and operators cause significant change in test results. A second operator tested one disk from the test set of five FTA cards using a different PCR thermocycler. Reproducibility was calculated as the percent agreement between the results of tests under different conditions.^13^

Repeatability and reproducibility were assessed for UCB and saliva spotted on FTA cards using six randomly selected cards for each specimen and running the same experiment as described for the venous blood. The considered cut off was 90% (Clinical Laboratory Improvement Amendments CLIA).

##### Stability of spotted FTA cards in local conditions

Each FTA card was stored in an individual plastic zip bag at room temperature, in a clean closet, whereas the extracted DNA was stored at −20°C. We randomly selected 15 FTA cards from study group 1.

The selected FTA cards were repeatedly tested every 6 months by FTA‐RFLP as described above. Thus far, we have completed testing after 4 years of conservation.

### 2.2 Data analysis

The SO‐PRC‐RFLP was considered as the gold standard to which results from the FTA cards were compared. The FTA‐RFLP method was compared to the gold standard regarding the cost and conditions. Sensitivity and precision were computed. Statistical analysis was performed with SPSS version 21. *Kappa* statistics was used to evaluate the agreement between two different testing methods.

We also assessed the stability of FTA‐spotted venous blood samples after 4 years of storage at room temperature.

### 2.3 Ethical compliance

This study was compliant to international ethics laws and regulations. Prior to the inclusion, parents and patients were fully informed and provided signed informed consent/assents. The study protocol was approved by the Ethical Committee of the Public Health School at the University of Kinshasa (ESP/CE/079/2016).

## 3 RESULTS

### 3.1 FTA‐RFLP on peripheral blood

**FIGURE 2:**
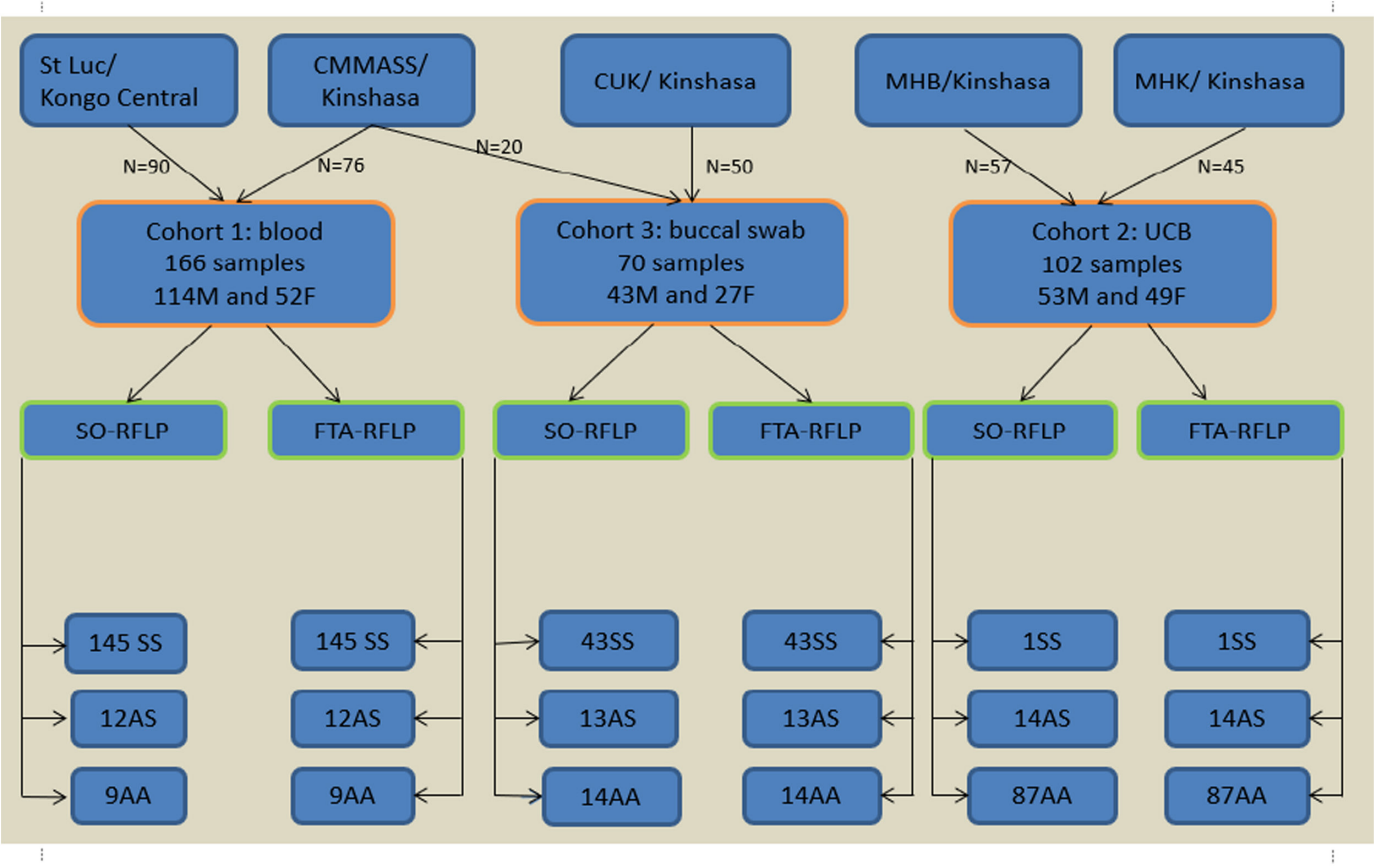
Study algorithm and results. The top row presents the five institutions where participants were recruited; the second row shows the composition and specimens for the three cohorts; the third row indicates the two testing approaches applied to each of the cohort. The distribution of the genotypes obtained by either of the testing approaches for each cohort is presented at the bottom of the figure

This test was performed on the 166 participants with known electrophoresis‐based hemoglobin profile recruited in Kinshasa and Kisantu. The FTA‐RFLP identified 145 homozygotes SS, 12 heterozygotes AS and nine homozygotes wild‐type AA. The SO‐RFLP, performed on DNA extracted from venous blood collected on EDTA tubes, showed the same results for each participant. Therefore, there was a perfect agreement between FTA‐RFLP and SO‐RFLP (*kappa* = 1; Figure 2).

### 3.2 FTA‐RFLP on umbilical cord blood

The 102 FTA cards containing UCB samples were tested by FTA‐RFLP and returned 1 SS, 14 AS, and 87 AA. Later, the genomic DNA extracted from the 102 UCB samples collected in the EDTA tubes was also tested by SO‐RFLP. This returned the exact same result for each participant, corresponding to a perfect agreement between the two techniques (*kappa* = 1; Figure 2).

### 3.3 FTA‐RFLP on buccal swab samples

The FTA‐RFLP test performed on swabs rubbed on FTA cards from 70 participants with known SO‐RFLP genotypes, identified 43 SS, 13 AS, and 14 AA. These results were in agreement with the profiles previously obtained by SO‐RFLP performed on DNA extracted from blood sampled from the same participants (Figure 2). Thus, there was a perfect agreement between swab‐based FTA test and blood‐based DNA test (*kappa* = 1). This validated the sensitivity and precision of the DNA‐based Sickle Cell Anemia diagnostic test on buccal swab samples.

### 3.4 Sensitivity and precision

The results from venous blood collected on FTA cards, UCB spotted on FTA cards and swabs rubbed on FTA cards, all returned identical results to the respective outcome obtained with the SO‐RFLP. No false positive or false negative result was observed. Therefore, the sensitivity as well as the precision was 1 for DNA‐based SCA test using either of those three specimens.

### 3.5 Repeatability and reproducibility

Intra‐ and inter‐run replicates returned the exact same profiles for each of the randomly selected individuals. Moreover, the change in the operator and the PCR thermocycler did not alter the outcome of the test.

### 3.6 Stability of spotted FTA cards in local conditions

**FIGURE 3:**
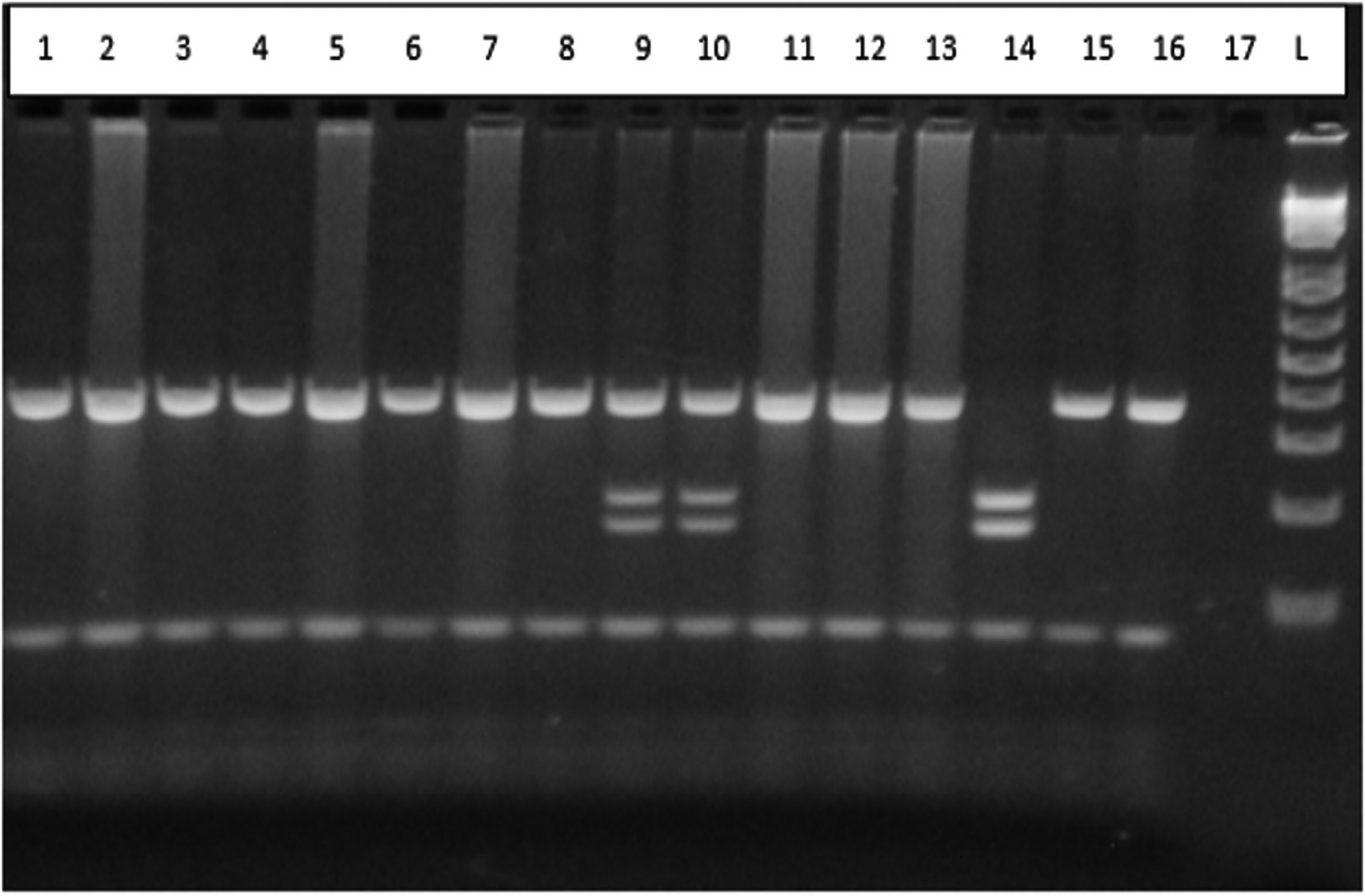
Restriction profile after 4 years of storage of some FTA Elute blood samples. Lane 1 to 8 shows homozygous mutation SS profiles, lanes 9 and 10 show heterozygous AS, lane 11 to 13 show homozygous SS, lane 14 represents wild‐type AA, lane 15 shows homozygous SS, lane 16 contains a positive control whereas lane 17 is the no DNA. The size ladder is on the L lane

The results of 4 years of storage indicated that the profile remained clearly recognizable, suggesting the DNA quality was not altered by the local storage conditions: shelve storage in a dust‐free closet at room temperature (between 25 and 35 Celsius degrees). The results remained identical over the years up to 4 years when the last test was performed (Figure 3).

### 3.7 Conditions and cost comparison

The conditions and the cost of both tests are presented in Table 2.

**TABLE 2:** Comparison of conditions and cost

	Salting out	FTA card
Condition		
Sample collection	EDTA tubes	FTA cards
Sample transport	RT	RT
DNA extraction	Yes	No
Storage	Freezer −20°C	RT
Turn‐around time (DNA extraction + PCR‐RFLP)	24 h + 5 h	5 h
Cost estimation		
Consumables for sample collection and DNA purification	2.3 €^a^	1.51€ (half a card/patient)
PCR reaction per sample	2 €	2 €
Restriction enzyme digestion	1.2 €	1.2 €
Gel electrophoresis	0.8 €	0.8 €
Equipment depreciation	0.40 €	0.10 €
Personnel cost^b^	4.85€	0.9 €
Total cost	11.55 €	6.51 €

Abbreviations: NA, not applicable; RT, room temperature.

^a^ Based on cost estimation from Chacon‐Cortes et al.^14^

^b^ Based on the average monthly salary of 500$ for a laboratory technician in DRC. The daily salary average is 25$ per day. The hourly salary is 25$/8 = 3.152 $. The hourly salary is based on the legal limit of 8 working hours a day. For the SO‐RFLP, the full run includes 16 samples. The hourly salary per sample is 3.125$/16 = 0.195$. For the 29 hours of the run, each sample costs 0.20$ × 29 = 5.8$ (4.85€) as personnel costs. For the FTA‐RFLP, the full run includes 14 samples. The hourly salary per sample is 3.1$/14 = 0.2$. For the 5 hours of the run, each sample costs 0.2$ × 5 = 1.0$ (0.9€) as personnel cost.

The cost of the SO method was based on estimation by Chacon‐Cortes et al.^15^ While the personnel cost for both SO‐RFLP and FTA‐RFLP was estimated according to the average monthly salary of a laboratory technician in DRC.

## 4 DISCUSSION

We conducted a prospective technical validation study aiming at implementing a DNA‐based test for SCA that is feasible in a resource‐limited setting such as the DRC, despite infrastructure, environmental and economic challenges. Among the challenges we wanted to overcome were (1) sample transfer conditions across a country that is as big as the Western Europe and (2) instability of electricity on which depend laboratory storage facilities. In addition, we wanted the test to be efficient on non‐invasive specimen such as umbilical cord blood and buccal swabs. Finally, we also wanted this test to remain within the cost range of the most used test, the hemoglobin electrophoresis. To the best of our knowledge, neither the DNA‐based SCA test nor the use of non‐invasive specimen is currently offered as first‐line test in routine for SCA diagnostic in Central Africa.

Due to the ease in sample collection, transport and handling, the use of DBS cards such as FTA cards is gaining in acceptance for genetic analysis in multiple fields including infectious diseases, agriculture, and pharmacogenetic.^16^, ^17^, ^18^, ^19^, ^20^, ^21^

The RFLP was previously identified as a reliable and cheap approach for DNA‐based diagnostic of SCA.^7^ However, users should note that this test cannot differentiate HbS from HbC since both mutations affect the same restriction site and result in the same profile on agarose gel electrophoresis. Also, this test is not designed for the identification of others mutations that may cause SCA phenotype, such as compound heterozygous HbS/βthal or HbS/HbO. Fortunately, such mutations are rare in the DRC and absent from this cohort (based on sequencing data, not included in this article).

The FTA cards were tested as an alternative to the EDTA tube sampling and DNA extraction by salting out. In this study, we obtained the same genomic profile using a 1.2 mm FTA disks or 1µl of DNA extracted by salting out from the same participant. We conclude that using FTA cards is technically as good as the extracted DNA for sickle cell testing by RFLP. The amount of material on a dry blood spot may vary from one individual to another. In this study, all participants had clear PCR and digestion bands on agarose gel irrespectively of their disease status. This suggests that a 1.2 mm disk of FTA card retains enough DNA sample to allow the test to be carried out without DNA extraction.

The value of the FTA‐based test was verified using two other specimens namely UCB and buccal swabs in comparison to genomic DNA extracted from peripheral blood. The UCB is an easily accessible material. This prevented us from sampling newborns by heel punctures. Therefore, this was the ideal and non‐invasive material to test the FTA‐based approach in newborns. Results from UCB samples on FTA cards proved to be very reliable. Considering that electrophoresis‐based diagnostic test for SCA is unreliable before the age of 3 months,^22^ the FTA‐based test may be offered as a confirmatory test for suspect electrophoresis‐based newborn screening test results or as a first‐line test in high‐risk couples. This is of high value when considering both the reported delay in diagnosis and the heavy burden of sickle cell in Sub‐Saharan countries. As previously reported,^23^ our study has proven that a buccal swab is a good alternative of DNA for a genetic test such as SCA diagnostic. Proposing such a non‐invasive approach may increase the acceptability of SCA tests and newborn screening.

The sensitivity and precision of FTA‐RFLP for either venous blood, UCB, or buccal swab were 100%, confirming that irrespectively of the specimen and the amount of DNA, FTA‐based test is reliable.

It is common to centralize genetic testing in a resource‐limited setting in order to ensure efficient resource sharing. The Center for Human Genetics of the University of Kinshasa is positioned to become the national reference center for molecular testing not only for SCA but also for various genetic tests. Whole blood, the most used material for genetic analyses, is a type of sample that can be stored only in certain conditions with respect to the cold chain and for a relatively short period. But in DRC, the unreliability of electricity supply and the lack of quick and safe sample transportation would not always guarantee an appropriate transport of whole blood samples to the laboratory. Moreover, transport from the collection site to the testing laboratory is laborious and requires an expensive investment for transport. Hence, it would be appropriate to use a consumable that can provide safe blood sample transportation, such as DBS cards.

The issue of power supply is critical for a laboratory storage facility or any laboratory storage in resource‐limited countries. Moreover, the storage capacity is often very limited, which might be a problem for a country with about 2 840 000 births per year including an estimate of 40000 SCA babies (1.4%) per year.^1^

Our results show that DBS cards can facilitate sample collection, ensure safer transportation of samples, shorten the turn‐around‐time by skipping the DNA extraction step and return reliable results even after several years of storage at room temperature.^24^, ^25^, ^26^, ^27^ In addition, based on the possibility of stacking the zip bags, it becomes possible for the biorepository to store an enormous amount of samples. This implies that a national screening program for SCA should consider using DBS cards or similar material for testing and for safe and long‐term storage of samples at a minimal cost.

The FTA‐RFLP was estimated to cost almost half of the SO‐RFLP (Table 2). This is mainly due to the fact that the SO‐RFLP is labor‐intensive and time‐consuming. This resulted in a high personnel cost that made a significant difference between the two approaches. Interestingly, the estimated cost for SO‐RFLP is also half of the price commonly charged in Kinshasa for the IEF. Hence, our proposed approach of FTA‐RFLP is not only more accurate than IEF, but it is also more efficient on challenging specimens such as UCB, it is suited for non‐invasive testing using a buccal swab, and, most importantly, four times cheaper than the IEF. Therefore, switching to DBS DNA‐based test as the confirmatory approach in the newborn screening program, and as first‐line test for SCA diagnostic in other aged groups will significantly reduce the financial burden while offering the highest accuracy. This approach can also be offered in mass campaigns as a non‐invasive method.

In addition, this test can be used as first‐line test for screening sickle cell trait in blood donors and blood donations. This will increase blood transfusion safety.^28^

## 5 CONCLUSION

The study aimed at implementing a DNA‐based SCA test in resource‐limited settings and evaluating the economic benefit. We have shown that this procedure is feasible in resource‐limited settings, retains high accuracy for various sample types, including venous blood, umbilical cord blood, and buccal swab, while remaining affordable. We recommend this test as a first‐line diagnostic test after transfusion as well as in blood donors and blood donations, and as second line for newborn screening.

## AUTHOR CONTRIBUTIONS

Prosper Lukusa, Koenraad Devriendt, Gert Matthijs and Aimé Lumaka designed the project and corrected the manuscript. Mamy Ngole, Paul Lumbala, and Gloire Mbayabo conducted patients’ recruitment. Mamy Ngole and Cathy Songo performed laboratory analysis under supervision of Valerie Race. Mamy Ngole prepared the manuscript; Prosper Lukusa, Koenraad Devriendt, Gert Matthijs, Aimé Lumaka, and Valerie Race edited the manuscript. All authors read and approved the final manuscript.

## Data Availability

We included all the data related to this research in the article. Moreover, all data are available from the corresponding author.
